# Mineralization and Preservation of an extremotolerant Bacterium Isolated from an Early Mars Analog Environment

**DOI:** 10.1038/s41598-017-08929-4

**Published:** 2017-08-18

**Authors:** F. Gaboyer, C. Le Milbeau, M. Bohmeier, P. Schwendner, P. Vannier, K. Beblo-Vranesevic, E. Rabbow, F. Foucher, P. Gautret, R. Guégan, A. Richard, A. Sauldubois, P. Richmann, A. K. Perras, C. Moissl-Eichinger, C. S. Cockell, P. Rettberg, Marteinsson, E. Monaghan, P. Ehrenfreund, L. Garcia-Descalzo, F. Gomez, M. Malki, R. Amils, P. Cabezas, N. Walter, F. Westall

**Affiliations:** 1 Centre de Biophysique Moléculaire, CNRS, Orléans, France; 2 Institut des Sciences de la Terre d’Orléans, UMR 7327, CNRS-Université d’Orléans, 1A Rue de la Férollerie, 45071 Orléans Cedex 2, France; 3 Institute of Aerospace Medicine, Radiation Biology Department, German Aerospace Center (DLR), Cologne, Germany; 4 UK Center for Astrobiology, School of Physics and Astronomy, University of Edinburgh, Edinburgh, United Kingdom; 5 MATIS - Prokaria, Reykjavík, Iceland; 6 Centre de Microscopie Electronique, Université d’Orléans, Orléans, France; 7 BioTechMed Graz, Graz, Austria; 8 Leiden Observatory, Universiteit Leiden, Leiden, Netherlands; 9 Instituto Nacional de Técnica Aeroespacial – Centro de Astrobiología (INTA-CAB), Madrid, Spain; 10 Universidad Autónoma de Madrid (UAM), Madrid, Spain; 11 European Science Foundation (ESF), Strasbourg, France; 12 University Regensburg, Department of Microbiology, Regensburg, Germany; 13 Medical University of Graz, Department of Internal Medicine, Graz, Austria

## Abstract

The artificial mineralization of a polyresistant bacterial strain isolated from an acidic, oligotrophic lake was carried out to better understand microbial (i) early mineralization and (ii) potential for further fossilisation. Mineralization was conducted in mineral matrixes commonly found on Mars and Early-Earth, silica and gypsum, for 6 months. Samples were analyzed using microbiological (survival rates), morphological (electron microscopy), biochemical (GC-MS, Microarray immunoassay, Rock-Eval) and spectroscopic (EDX, FTIR, RAMAN spectroscopy) methods. We also investigated the impact of physiological status on mineralization and long-term fossilisation by exposing cells or not to Mars-related stresses (desiccation and radiation). Bacterial populations remained viable after 6 months although the kinetics of mineralization and cell-mineral interactions depended on the nature of minerals. Detection of biosignatures strongly depended on analytical methods, successful with FTIR and EDX but not with RAMAN and immunoassays. Neither influence of stress exposure, nor qualitative and quantitative changes of detected molecules were observed as a function of mineralization time and matrix. Rock-Eval analysis suggests that potential for preservation on geological times may be possible only with moderate diagenetic and metamorphic conditions. The implications of our results for microfossil preservation in the geological record of Earth as well as on Mars are discussed.

## Introduction

Redrawing the history of early life on Earth requires being able to assess if microstructures present in the oldest terrestrial rocks are of biological origin or not. Such assessments are still very challenging mainly due to the degradation of microbial remains during diagenesis and to microbial-like morphologies abiotically produced. Several Archaean rocks could nevertheless be described as ancient unambiguous biological systems, representative of the early-Earth fossil record, like strata of South Africa and Australia containing evidence of phototrophic^1–6^ and heterotrophic microbial.

To better understand the processes leading to microfossil formation and preservation, artificial mineralization of microorganisms, also called fossilisation, was first undertaken by Oehler and Schopf with the silicification of Cyanobacteria^7^, followed by the mineralization of various eukaryotic and bacterial models^8, 9^ and later of natural communities^10^. The approach has since been extended to phylogenetically diverse microorganisms, notably to extremophiles, such as thermophilic Bacteria^11–13^ or hyperthermophilic Archaea^14, 15^ and their viruses^16^. Although these studies used different environmental protocols (nature and concentration of the silicification agents, temperature or duration), they reported that preservation by mineralization strongly differs from one microbial group to another, depending on their physiology (extracellular structures)^17^ and metabolism (metal reduction or oxidation)^18^. For a review of biomineralization see Li *et al*.^19^.

However, the embedding of cells in a mineral matrix should not be confused with the preservation of cells over long geological timescales. In natural environments, the first step of microbial embedding in a mineral casing, lasting from hours to months, needs to be followed by cementing of the whole system, including both sediments and mineralized microorganisms, in order to preserve traces of life over geological time lasting from millions to billions of years. More recent studies have thus focused on “artificial ageing” of organic matter and cells to better understand changes in their biosignatures and subsequent detectability in such contexts^19–24^. In this paper we term “mineralization” the precipitation of minerals around and inside cells and their embedding in a mineralogical matrix.

Although artificial mineralization can also be applied to answer astrobiological questions, it has still not been used in the search for traces of life in extraterrestrial samples, notably in Martian rocks. However, it is now clear that environmental conditions on early Mars were very different to those of present-day Mars, allowing serious consideration of its past habitability and the emergence of life^25–27^. This is especially true for the Noachian period, associated with intense volcanic activity supplying heat and chemical energy to the planet, and from which several studies documented widespread liquid water^28, 29^ as well as the possibility for past habitable fluviolacustrine environments^30^. Major changes in environmental conditions may have progressively challenged the potential Martian biological systems and thus, different scenarios can be envisaged for the history of Martian life^31^.

Drilling the Martian subsurface for past life detection, as scheduled with the European Exomars 2020 mission will provide important information about or evidence, especially organochemical, of past life on Mars. In the meantime, astrobiologists can use terrestrial analogues to study past Martian habitability^32^.

In this context, the 4 year-long (2014–2017) European MASE project (Mars Analogue for Space Exploration) uses analogue environments to assess the habitability of Mars and the detection of life on the planet. For that, a better of understanding biomarker preservation during microbial mineralization and fossilisation is essential. Several strains were isolated from the Icelandic Graenavatn lake, an acidic (pH3), cold and oligotrophic volcanic crater lake. The polyextremotolerant bacterium *Yersinia intermedia* MASE-LG-1, hereafter named *Yersinia*, was used in this study since it revealed a strong tolerance to diverse Mars-like stresses such as low pressure, ionizing radiation, varying temperature, osmotic pressure and oxidizing chemical compounds and represents thus a relevant model for mineralization.

In this paper, we report on the early mineralization of *Yersinia intermedia* MASE-LG-1 in silica and gypsum, two minerals commonly reported on Mars, in cold and anoxic conditions, similar to Martian conditions. We studied the effect of physiological status on mineralization by exposing *Yersinia intermedia* MASE-LG-1 to two stresses thought to have increased during Mars history, desiccation and radiation. The mineralization process was studied using microbiological (microbial viability), morphological (scanning and transmission electron microscopy), biochemical (GC-MS, Microarray immunoassay and Rock-Eval) and spectroscopic (FTIR and RAMAN spectroscopy) methods. Due to strong limitations to extrapolate short-duration experiments to the preservation of biosisigatures on geological times, we discuss the possibility (or potential) of mineralized *Yersinia intermedia* MASE-LG-1 cells for further long-term fossilisation.

## Materials and Methods

### Isolation of the *Yersinia intermedia* MASE-LG-1 strain

The bacterial strain was isolated from sediments in the Icelandic Graenavatn lake (63°.88′44″N; 22°05′40″W) during a MASE field campaign led in September 2014. Graenavatn lake is acidic (pH3), cold (4 °C) and oligotrophic. Isolation was strictly anaerobically performed in MASE I medium. Briefly, this medium consists in NH_4_Cl (0.5 g.l^−1^), NaHCO_3_ (0.2 g.l^−1^), NaH_2_PO_4_ (0.06 g.l^−1^), Cystein-HCl (0.5 g.l^−1^) gased with CO_2_/N_2_ 20/80. It was supplemented with KNO_3_ (0.01%) and C-Org-Mix (0.01%). Following enrichments in a range of media relevant to this environment, this organism finally was isolated, showing optimal growth at pH7 and 30 °C.

### Exposure to Mars-like conditions

Exposure to Mars-like stresses was carried out at the German Aerospace Center (DLR) in Cologne, Germany. To expose our model to a real stress, the intensity of stress has to (i) not kill all cells and (ii) lead to a real and strong microbial response. To satisfy these two points, we exposed the model to its LD_50_ value for radiation (dose for which 50% cellular viability is reached). Briefly 500 µl of an anaerobic culture of overnight-grown *Yersinia* grown overnight in MASE I medium (30 °C, pH7, cell density ~2.10^7^cells.ml^−1^) were desiccated on glass slide for one day under anaerobic conditions. The slides with desiccated cells were then transferred into vials for irradiation by X-rays. Tolerance of *Yersinia* to radiation was first tested in order to apply the radiation dose corresponding to its LD_50_, estimated at 40 Gy. A dose of 4.42 Gy.min^−1^ was thus applied during 9 minutes and 17 seconds. After exposure, desiccated and irradiated cells on glass slides were immediately resuspended in 10 ml of the mineralization solutions.

### Mineralization of the *Yersinia* strain

To induce the mineralization of the non-stressed *Yersinia* strain, a preculture was grown overnight in MASE I medium (30 °C, pH7) and 5 ml of the exponential phase culture (~2.10^7^cells.ml^−1^) were transferred into 45 ml of the mineralization solutions within an anaerobic chamber. Solutions were made anoxic by bubbling with N_2_/CO_2_ (80/20 vol/vol). The silica solution consisted of 500ppm silica using Tetraethylorthosilicate (TEOS, Sigma Aldrich) in water and with 30 g.l^−1^ of sea salts (Sigma Aldrich) to mimic saline and briny conditions, more similar to what could be encountered by cells in natural environments. Although no data exists on the nature and concentration of organics on Mars during the Noachian, it is considered that organic matter on Mars was globally rare at that time and thus, no carbon source was added in vials. The gypsum solution consisted in CaSO_4_ in saturated concentration (2.1 g.l^−1^) in water supplemented with 30 g.l^−1^ of Sea salts. To prevent contamination, Milli-Q water filtered through a 0.2 µm filter. Identical solutions were used for stressed *Yersinia*. Anoxic vials were then stored for 6 months at 4 °C during the mineralization process.

### Viability of the strain during mineralization

The viability of the *Yersinia* strain during mineralization was monitored in MASE I medium using the Most Probable Number (MPN) method. For that, the cell density of the preculture used for mineralization was determined by cell counting with a Thoma chamber and the positive growth of serial dilutions (from non-diluted to dilution 10^−8^) was checked by optical microscopy.

Live/dead staining was also performed to distinguish cells with possible disrupted membranes from cells with probable intact membranes using LIVE/DEAD^®^ Bac Light^TM^ BacterialViability Kits (ThermoFisher Scientific), according to manufacturer’s instructions. Stained cells were observed by epifluorescence microscopy with a Zeiss Axiovert 200 microscope, equipped with an Hg-lamp. Acquisitions were made with the AxioVision software.

### Electron microscopy

Samples for electron microscopy analyses were fixed in paraformaldehyde (final concentration 2.5%, w/v), washed 3 times for 15 min in sodium phosphate buffer (pH 7.4) and filtered on a 0.2 µm filter.

For Transmission Electron Microscopy (TEM), filter sections were placed into microporous ceramic capsules for dehydrating using increasing ethanol⁄distilled water solutions (25%, 50%, 75% v/v ethanol 15 min each and 100% ethanol 1 h) followed by an ethanol⁄acetone series (25%, 50%, 75% v/v acetone 15 min each and 100% acetone 1 h). Contrast was increased by osmium tetroxide coloration using an OsO_4_ 4% aqueous solution (Electron Microscopy Sciences). The upper part of the filter was gold-coated prior to dehydrating in order to distinguish the upper and the lower parts of the filters during TEM observations. The samples were then prepared for ultrathin sectioning by embedding in TAAB 812 Resin (TAAB Laboratories, UK), with a series of acetone ⁄ resin mixtures in the proportions 3:1, 1:1 and 3:1, followed by one bath in 100% resin (overnight). The resin blocs were finally left to harden at 60 °C in a last 100% resin bath. Ultrathin sections were made using a Diatome diamond knife mounted on a Reichert ultramicrotome and placed on copper TEM grids. Observations and analysis were made with a Philips CM20 Transmission Electron Microscope, equipped with an EDX detector (Oxford Instruments).

For SEM, dehydrated samples were gold coated and finally observed and analyzed with a Hitachi S4500 Field Emission Gun Scanning Electron Microscope, equipped with an EDX detector (Oxford Instruments).

### Spectroscopic analyses

Samples for RAMAN and FTIR (Fourier-Transform Infrared) were washed in Milli-Q water after 3 washing cycles. For RAMAN, the last pellet was suspended in 200 µl of Milli-Q water and the cell suspension was air-dried onto a CaF_2_ slide. RAMAN analyses were made with a WITec Alpha500 RA Raman spectrometer equipped with a green laser (Nd:YAG frequency doubled, wavelength 532 nm). Two cumulative spectra were acquired for 10 to 30 minutes depending on the sample. The laser power was set between 5 and 10 mW.

For FTIR, washed cells were lyophilized and incorporated in dried potassium bromide and then pressed under standards pressure (nine tons) to produce tablets. FTIR measurements in the range 650–4000 cm^−1^ were recorded using a Thermo Nicolet 6700 FT spectrometer equipped with a Deuterated Triglycine Sulfate (DTGS) detector and a Nicolet Continuum microscope. Analyses were performed in transmission mode. Each spectrum was the average of 256 scans collected at 2 cm^−1^ resolution.

### Geochemistry and fatty acid analyses

Rock-Eval pyrolysis (aiming at determining the organic content, the oil potential and the nature and maturity of kerogens within rocks) was carried out on ~75 mg lyophilized cells with a Turbo Rock-Eval 6 pyrolyzer. An IFP 160000 standard was used to calibrate the measurements. The pyrolysis program was as follows: 2 min at 200 °C, raised to 650 °C at 30 °C·min^−1^. The oxidation phase (air stream) began with an isothermal stage at 400 °C, which was then increased to 650 °C (30 °C·min^−1^) and held for 5 min at this temperature.

For GC-MS analyses, fatty acid extraction was carried out after lyophilization and treatment of cells to acidic hydrolysis in HCl 5N for 20 h at 120 °C. Neutralization was then done with NaOH 5 N. SOLID Phase Extraction (SPE) was used with NH2 columns (Supelco) to retain organic molecules. Briefly, the columns were activated with 5 ml H_2_O prior to passing the samples washed with 5 ml H_2_O. Molecules were removed using 2.5 ml ether:formic acid 9:1 (vol:vol) and finally with 5 ml methanol. After evaporation of the solvents with a rotavator, silylation of the molecules was achieved in 2 × 500 µl of pyridine with brief desorption of molecules by ultrasonication in 300µl N-tert-Butyldimethylsilyl-N-methyltrifluoroacetamide (MTBSTFA) for 1 h at 60 °C. Pyridine was then evaporated by nitrogen flow and replaced by 150 µl heptane. For quantification, bisphenol A was added as an internal standard before extraction to estimate extraction yields and a second standard, 5-α-cholestane, was also added just before GC-MS injection.

Injections were made on a Trace GC Ultra gas chromatograph (GC) coupled to a TSQ Quantum XLS mass spectrometer equipped with an AS 3000 autosampler (both from Thermo Scientific). The GC was fitted with a Thermo Trace Gold TG-5 MS capillary column (60 m, 0.25mm internal diameter, 0.25μm film thickness). The temperature of the column was held at 40 °C for 1 min, and then increased from 40 to 120 °C at 30 °C.min^−1^, and then from 120 to 300 °C at 3 °C.min^−1^ with a final isothermal hold at 300 °C for 30 min. 2 μL of sample were injected in splitless mode at 280 °C. Helium was the carrier gas (1 ml.min^−1^). The mass spectrometer was operated in EI mode at 70 eV, from m/z 50 to 600. Spectra were analyzed with the Thermo Xcalibur 2.2 SP1.48 software.

### SOLID-Chips

Mineralized *Yersinia* cells were analyzed with a sandwich microarray immunoassay containing 168 antibodies against whole microbial cells of bacterial and archaeal groups, extracellular polymers, environmental extracts from terrestrial analogues of Mars and Europa Moon, two planetary bodies of astrobiological interests, and proteins/peptides from well-conserved anaerobic metabolic pathways.

The sandwich immunoassay consisted on an “*antibody*-*antigen*-*labelled antibody”* bound with extracted epitopes from 5 ml of treated and non-treated *Yersinia* cultures. The extraction were carried out by sonication (3 cycles of 30 seconds at maximum amplitude on ice) in buffer extraction (NaCl 0,3 M, Tris-HCl 0,4 M pH = 8, 0.1% Tween 20). Sonicated samples were filtered through a 5 µm nylon membrane to remove possible minerals and coarse materials.

The microarray was firstly blocked with 2% and 5% BSA (bovine serum albumin) in Tris-HCl buffer to minimize unspecific joints. The samples were then incubated for 1 h at room temperature to enable interactions between antibodies and the samples. A further washing step was done with buffer to remove excess of sample and to prepare the microarray for 1 h incubation with a labeled Alexa Fluor mix. The excess of labeled antibodies was washed and the slide was scanned by a red laser (635 nm). A highly sensitive CCD detector captured the fluorescent signals to generate an image. The intensity of the spots fluorescence of this image was analyzed and quantified with GenePix Pro software (Molecular Devices, CA, USA).

Samples and negative controls (buffer only without sample) were simultaneously analyzed. The spots intensities in the negative control were used to set a fluorescence control value. The final fluorescence of each antibody spot was calculated by subtracting the basal fluorescence in the control and considering as positives those exceeding 2.5 fold the control value.

## Results

### Electron microscopy

Observation of *Yersinia* cells under microscopy revealed that they consist in single, gram negative (after standard gram staining and optical microscopic observations), rods of 1.2 ± 0.2 × 0.6 ± 0.1 µm in size (n = 20), which did not form aggregates and which present a flagellar motility, although its location on cells could not be determined. No notable secreted materials, such as extracellular polymeric substances (EPS), were observed. To study the mineralization of cells we made SEM and TEM observations and determined the EDX spectra of cells during mineralization.

**Figure 1:**
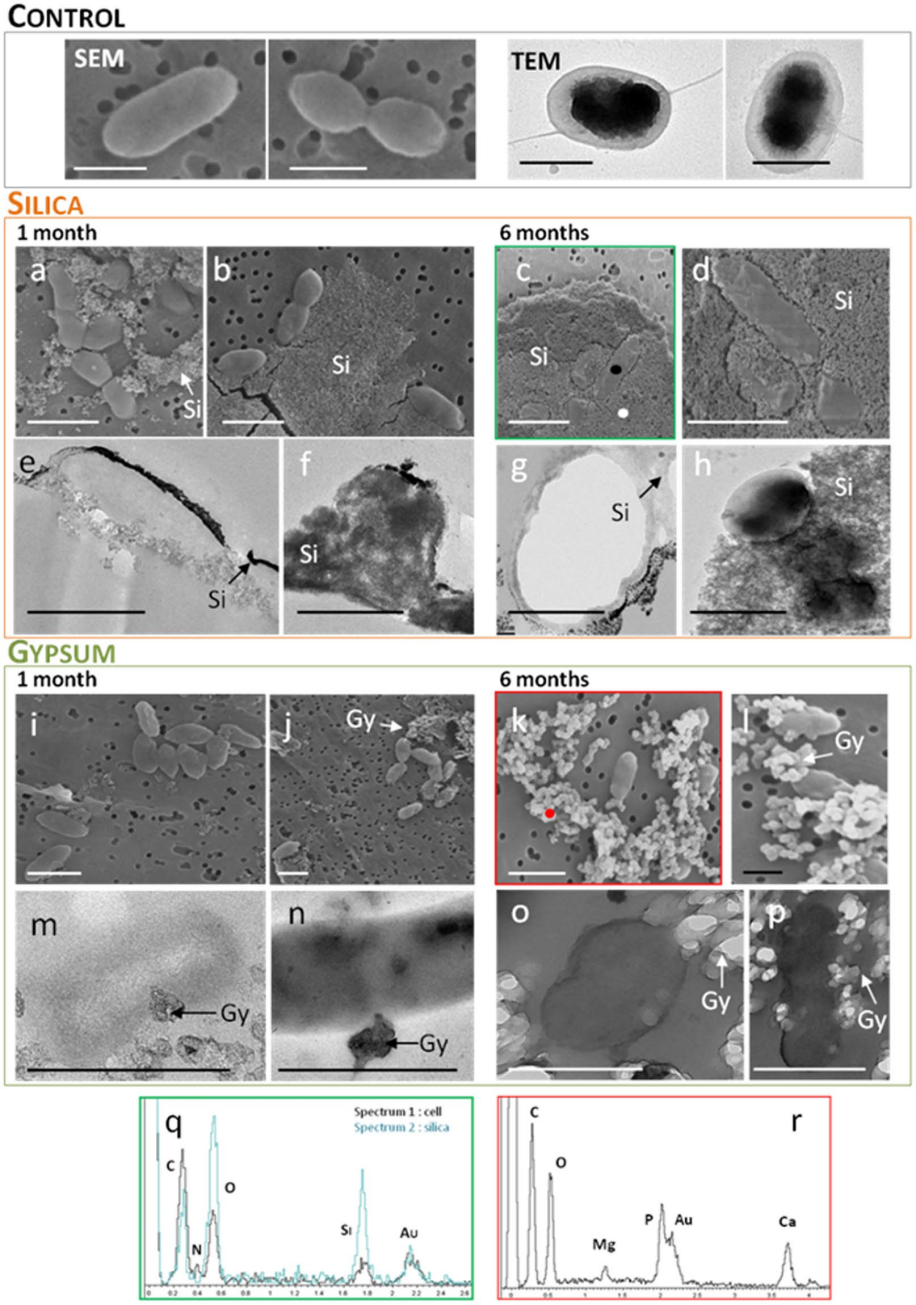
SEM and TEM images of non-mineralized *Yersinia* cells (upper panel in black) and after mineralization, either in silica (medium panel in orange) or in gypsum (lower panel in green). (**a**,**b**) SEM after 1 month, (**c**,**d**) 6 months and (**e**,**f**) TEM after 1 month, (**g**,**h**) 6 months in silica. (**I**,**j**) SEM after 1 month, (**k**,**l**) 6 months and (**m**,**n**) TEM after 1 month, (**o**,**p**) 6 months in gypsum. Scale bar: 1 µm. The occurrences of silica (Si) and gypsum with other sulfate compounds (Gy) is indicated. The EDX spectra of image C (**q**) is framed in green and the location of EDX analyses is indicated by black and white dots for cells and silica respectively. The EDX spectra of image K (**r**) is framed in red and location of analyze in indicated by the red dot.

In the silicification experiment, cells interacted immediately with the mineral when transferred to the mineralization solution since precipitation of silica around cells was observed in the first days of the experiment (Supplementary Figure 1). The texture of the mineral was typical of fine-grained amorphous silica (Fig. 1). Due to the very low metabolic activity of cells (no carbon source in the mineralization solution), it is likely that silica precipitation occurs passively due to the reactivity of functional groups of the outer membrane (sulfate, phosphate and carboxyl groups of lipopolysaccharides and membrane proteins) to SiOH monomers.

After one month of mineralization, we could observe cells either partially or totally embedded in silica (Fig. 1). Some cells were also free of minerals, revealing that mineralization is heterogeneous at the population scale, and this was also observed after 6 months (Supplementary Figure 1). No change in cell morphology was observed during mineralization, neither with silica and gypsum, nor with stress exposure, although a reduction of cellular size could be expected due to the starvation and metabolic constraints in the mineralization vials (no C sources). Silica precipitation was only observed on the outside of the cells and, even after 6 months, no evidence for silicification inside *Yersinia* cells could be observed in TEM sections (Fig. 1). This suggests that silica precipitates were constrained to the extracellular compartment and could not enter into cells at least during these 6 months. Microbial silicification increased with time and was highest after 6 months, as documented by the frequency of large, plate-like deposits of silica with embedded cells observed by SEM and TEM. The thickness of the silica layer around cells was strongly variable, ranging from few nanometers to several micrometers (Fig. 1).

Silicification of *Yersinia* cells exposed to desiccation and radiation gave similar results, *i*.*e* silicification was rapid, heterogeneous at the population scale and only occurred at the outside of cells.

Cells were distinguished from minerals based on their EDX spectra, showing a specific nitrogen signal as well as high levels of carbon and oxygen, indicative of organic matter. On the contrary, the EDX spectrum of silica precipitates only showed silica and oxygen while the weak carbon peak in some of the spectra was attributed to the carbon conductive adhesive tapes used in our experiment (Fig. 1).

The mineralization of *Yersinia* in gypsum was very different in both quantitative and qualitative aspects. Precipitation of the mineral occurred much more slowly, as indicated by the small amount of minerals observed after 1 month under electron microscopy (Fig. 1). They were more frequent only after 6 months. The texture of these minerals was also different to that of silica and only interacting very locally with cells compared to the large, plate-like deposits of silica, which could fully covered cells. It is likely that the entire embedding of cells by minerals requires a much longer mineralization process in our conditions. As observed for silicification, no change in cell morphology was observed with time and the occurrence of minerals was restricted to the outside of cells. EDX spectra of the mineral precipitates revealed the presence of elements, such as Mg and Ca, both coming from the mineral/seawater solution. Mg can substitute for Ca in gypsum (CaSO_4_·2H_2_O) but other minerals may have coprecipitated, such as, calcium phosphate, magnesium sulfate and/or magnesium phosphate (Fig. 1).

### Microbial viability

From a microbiological point of view, the mineralization of cells can be viewed as a chemical stress rather than synonymous with microbial death, as suggested by the induction of specific genes and physiological adaptation to silicification^33, 34^. We studied the viability of the *Yersinia* population during mineralization by the Most Probable Number (MPN) method. This approach revealed that 1% to 0.1% of cells were viable compared to the non mineralized control, corresponding to 10^4^ to 10^5^.ml^−1^ cells (10^6^ cells.ml^−1^ in the mineralization vials, Table 1). These results were also confirmed by Live/Dead staining (Supplementary Figure 2), indicating that cells with intact membranes (green fluorescence) were frequent, although we have to keep in mind that silicification can change membrane properties and thus the entry of molecular probes into cells. The viability of *Yersinia* is in agreement with our observation that not all cells are embedded in minerals and with previous the description of cyanobacterial viability and even activity during silicification^13^. This highlights that, although metabolically inactive, cells embedded in minerals can keep their cellular integrity and their molecular machinery intact, enabling them to resume normal cell activity when more favourable environmental conditions are encountered and when the silica is dissolved.

**Table 1:** Viability of *Yersinia* populations, exposed or not to a preliminary stress, after 1 and 6 months of mineralization, either in silica or in gypsum.

No stress					Desiccation + X-rays				
t_0_	1 month		6 months		t_0_	1 month		6 months	
	SiO_2_	CaSO_4_	SiO_2_	CaSO_4_		SiO_2_	CaSO_4_	SiO_2_	CaSO_4_
100%	10%	1%	0.10%	0.01%	10%	1%	0.10%	0.01%	0.01%

### Spectroscopic analyses

**Figure 2:**
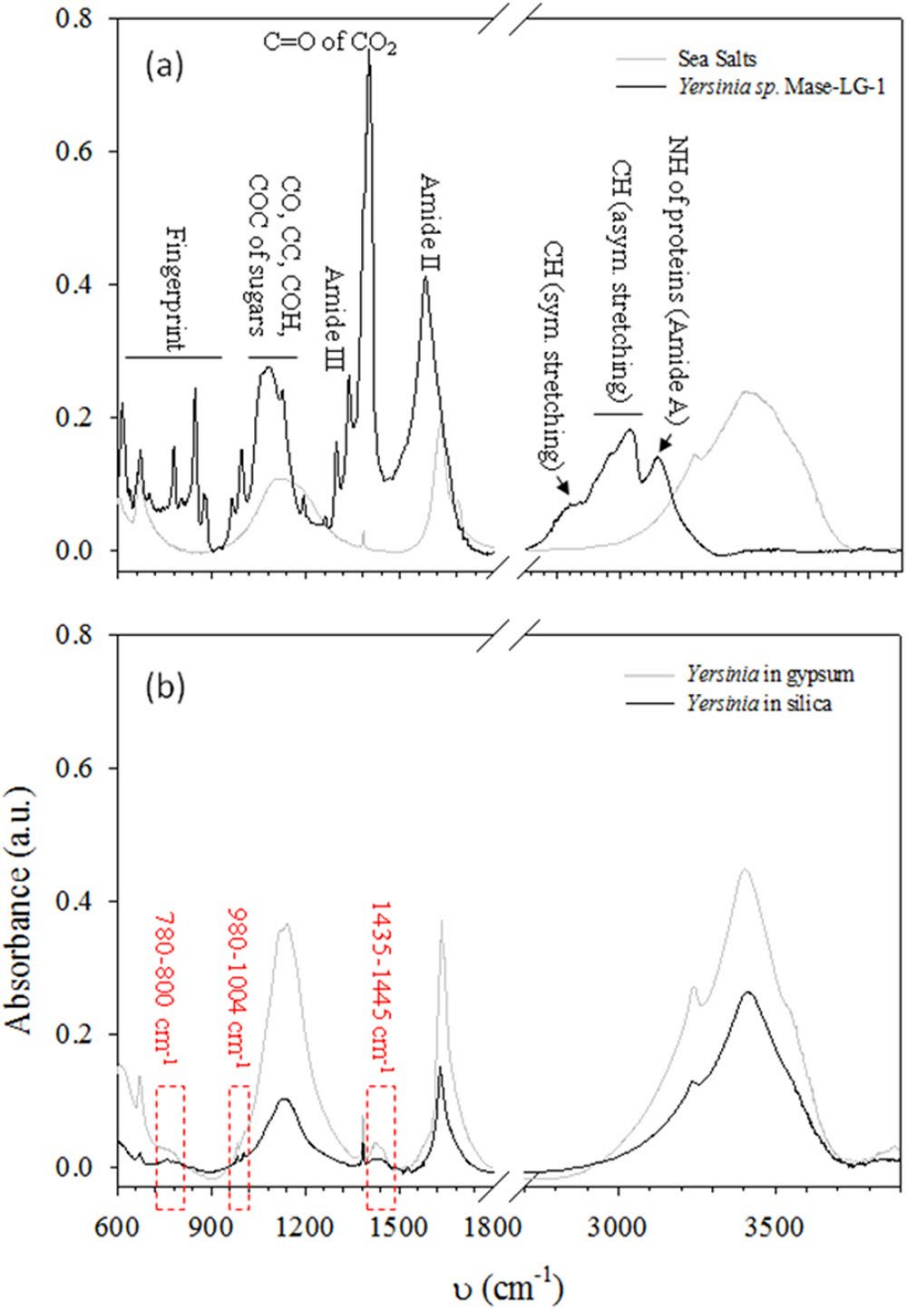
FTIR spectra of mineralized and non-mineralized cells. (**a**) FTIR spectra of controls, either positive with fresh *Yersinia* cells (black line), or negative for sea salts + gypsum + silica (grey line). (**b**) FTIR spectra of *Yersinia* after 6 months mineralization either in silica (black line) or in gypsum (black line). The three regions without absorption features in the negative controls are highlighted in red.

**Figure 3:**
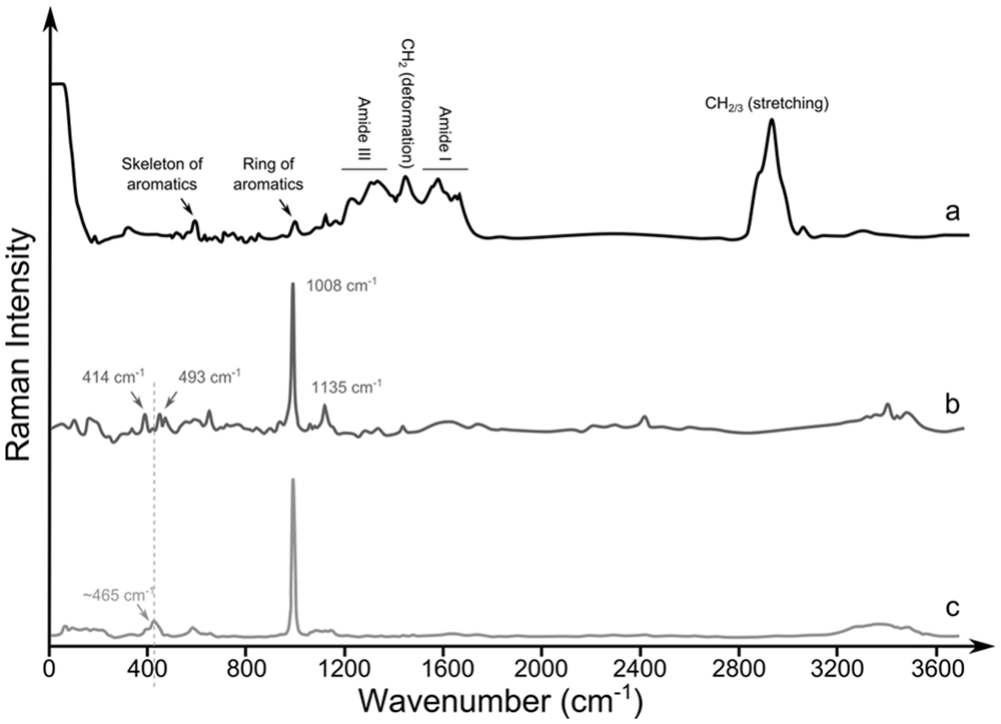
RAMAN spectra of mineralized and non-mineralized *Yersinia* cells obtained with a 532 nm laser. (**a**) RAMAN spectra of fresh *Yersinia* cells (black line), (**b**) RAMAN spectra of *Yersinia* after 6 months mineralization in gypsum (dark grey line) and (**c**) after 6 months of silicification (light grey line). The main peaks for biological molecules (**a**), gypsum (**b**) and silica (**c**) are indicated. The presence of gypsum signals in (**c**) reveals that the sea salts added to the vials can precipitate abiotically as sulfate minerals. Two cumulative spectra were acquired during 30″ each for (**a**) and during 10″ each for (**b**) and (**c**). The dotted line highlights that the 465 cm^−1^ peak of silica in (**c**) is different from the 414 and 493 cm^−1^ peaks in (**b**).

Vibrational (*i*.*e* RAMAN and FTIR) spectroscopy is a non-destructive and non-invasive method which provides information about the molecular composition and structure within a sample. It has been widely used in astrobiology to characterize either minerals or organic matter^6, 35–37^ and the Raman Laser Spectrometer (RLS) is an important part of the payload of the 2018 ExoMars rover^38^. To investigate whether vibrational signals from *Yersinia* remained detectable after mineralization, we analyzed non-mineralized (control) and mineralized cells using RAMAN and FTIR spectroscopy (Figs 2 and 3).

FTIR spectra of the “negative control” (sea salts + silica + gypsum) gave peaks at 3200–3600 cm^−1^ (OH stretching) and at 1140 cm^−1^, common to various sulfate minerals, such as CaSO_4_ 2H_2_O, MnSO_4_ 2H_2_O or K_2_SO_3_ 2H_2_O^39^. A peak at 1630cm^−1^, more specific to gypsum^39^, was also present (Fig. 2a). In agreement with the EDX spectra obtained with gypsum (Fig. 1r), these results suggest that several minerals, notably gypsum, may have coprecipitated around cells.

On the contrary, FTIR results of the “positive control” (non-mineralized cells) were typical of biological spectra already reported (Fig. 2a)^21, 40^. For example, the CH_2_/CH_3_ of fatty acids were represented by features in the 2900–300 cm^−1^ (CH asymmetric stretching), by the peak at 2880 cm^−1^ (CH symmetric stretching) and 1385 cm^−1^ (CH bending). Protein signals were also present, corresponding to peaks at 3150 cm^−1^ (NH stretching of amide A), 1580 cm^−1^ and 1340 cm^−1^ (respectively vibrational modes of amide II and amide III). Other major signals included the peak at 1400 cm^−1^ (C = O stretching of COO^−^), the region at 1050–1150 cm^−1^ (CO and CC stretching of carbohydrates) and the “fingerprint region” at 600–900 cm^−1^ ^40^.

FTIR analyses of cells after 6 months mineralization revealed that most of these signals were lost. However, when compared to the spectrum of the negative control, our samples showed 3 noticeable absorption features: (i) 1435–1445 cm^−1^, attributed to CH deformation of CH_2_, (ii) peaks at 980 and 1004 cm^−1^ which could represent CO, CC, COH or COC of carbohydrates and (iii) region 720–800 cm^−1^ corresponding to the “fingerprint region” (Fig. 2b).

This shows that even when largely modified, the FTIR spectra of *Yersinia* after mineralization are still characterised by specific signals of biological origin which could be detected in our experimental conditions.

Similar to the FTIR spectra, the RAMAN spectra obtained with fresh cells show the typical peaks of biological molecules (Fig. 3a). The highest peak was attributed to the stretching vibration of CH_2_/CH_3_ around 2935 cm^−1^ and 2970 cm^−1^. Another CH_2_ peak was represented by the deformation around 1450 cm^−1^. Raman modes of proteins were represented by the amide I (1650cm^−1^) and the amide III (1330 cm^−1^) peaks, as well as by peaks for the ring (1005 cm^−1^) and the skeleton (600 cm^−1^) of aromatic aminoacids.

In contrast to the FTIR results, RAMAN signals of biological molecules could not be detected after 6 months of mineralization. Spectra were only characterized by peaks corresponding to sulfate minerals, such as gypsum (414 cm^−1^, 493 cm^−1^, 1008 cm^−1^ and 1135 cm^−1^) and to SiO_2_ (463 cm^−1^) in the case of samples used for silicification^41^ (Fig. 3b and c).

### Biomarker detection with SOLID-Chips

The possibility to detect traces of life *in situ* directly during field campaigns is of great interest for future astrobiological missions. The SOLID-Chip technology was developed to fulfill this requirement, by the detection of biomarkers in the form of universal antigens^42^. The use of these systems, based on sandwich microarray immunoassays leading to fluorescence emission, enabled successful detection of microorganisms and preserved organic matter in acidic, arid and oxidizing environments like the Atacama Desert or Rio Tinto^43, 44^. However, this technology has still not been used in the detection of microbial fossils. To investigate whether *Yersinia* biomarkers remained detectable after 6 months of mineralization, we used the SOLID-Chip system focusing on general antibodies, since highly conserved (“universal”) proteins/peptides are more likely to be detected than group-specific proteins/peptides.

**Figure 4:**
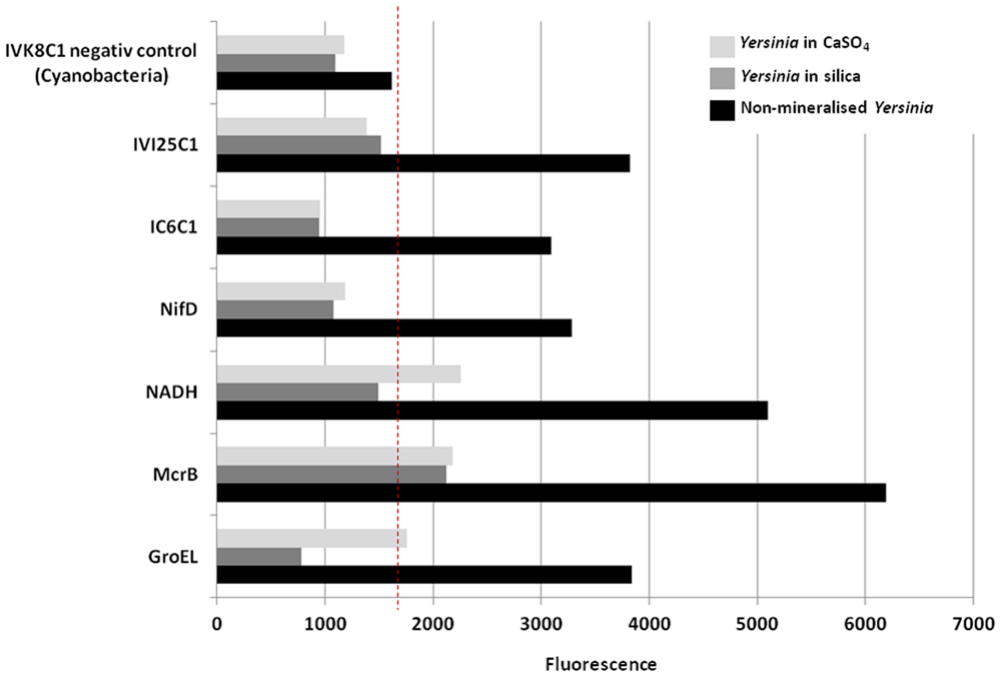
Bar diagram showing the fluorescence emission of general antibodies directed to the universal, either with non-mineralized *Yersinia* (black bars), with *Yersinia* 6 months in silica (dark grey bars) or 6 months in gypsum (light grey bars).

The results showed that, compared to non-mineralized cells, mineralized *Yersinia* cells were associated with a loss of fluorescence in both silica and gypsum (Fig. 4). The fluorescence of mineralized cells was lower than the background signal defined by the negative control (cyanobacterial antibody), whereas in all cases, non-mineralized cells strongly exceeded it. This revealed that the detectability of universal proteins/peptides was inhibited by mineralization. In some cases (NADH-synthase and GroeL proteins), a higher decrease in fluorescence was observed with silica than with gypsum, and may be explained by the greater degree of precipitation of silica around cells, as observed with electron microscopy (Fig. 4). However, even though embedding of cells in minerals is a likely explanation, we cannot exclude the possibility of interactions between minerals and the SOLID-Chip system, as well as changes in three-dimensional structures (or unfolding) of proteins during the mineralization process and sample storage with time, leading to the non-recognition of antibodies towards their specific epitopes. Regardless of these reasons, our results showed that long before cell preservation over billions years, the rapid mineral embedding of cells prevents biomarker detection using the SOLID technology.

### Rock-Eval

To investigate the potential of mineralized *Yersinia* to be preserved over long geological timescales, we studied the properties of its insoluble organic matter by Rock-Eval by determining in an inert atmosphere the quantity of released material from most volatile (S1) to most refractory (S4) fractions during heating, thus providing valuable information on the degree of maturity of organic matter. The results obtained with *Yersinia* mineralized in silica and gypsum were comparable and presented in Table 2.

**Table 2:** Main thermal maturity properties of the organic carbon in samples after 6 months mineralization in silica or in gypsum, as determined by Rock-Eval analysis.

	TOC (%)	S1 (mg.g^−1^)	S2 (mg.g^−1^)	S3 (mg.g^−1^)	S4 (mg.g^−1^)	T_peak_
*Yersinia* in silica (6 months)	0.31 ± 0.03	0.52 ± 0.06	1.91 ± 0.27	3.15 ± 0.05	0.60 ± 0.24	448 °C
*Yersinia* in gypsum (6 months)	0.35 ± 0.03	0.53 ± 0.06	1.93 ± 0.27	3.10 ± 0.05	0.65 ± 0.24	452 °C

Total Organic Carbon (TOC) is given in percent. S1 represents free hydrocarbons, S2 the hydrocarbons generated during heating, S3 the CO_2_ released by thermal breakdown of molecules and S4 the refractory residual carbon. T_peak_ represents the temperature leading to the maximum detection of materials.

The Total Organic Carbon content (TOC) was low (0.31%) and mostly represented by pyrolysable carbon (S1 + S2 + S3 fractions, ~90% TOC) and not by residual carbon level (S4 fraction, ~10% TOC). Free hydrocarbons (S1) represent a minor part of insoluble organic matter (0.52 mg.g^−1^ sample) whereas hydrocarbons generated during heating (S2), representing more mature carbon molecules, are more abundant (1.91 mg.g^−1^ sample), which is in agreement with a T_peak_ of 448 °C (the temperature corresponding to maximal release of materials). The level of CO_2_ released by thermal breakdown of molecules (S3) also indicates that more refractory compounds are present. However, the most refractory fraction of insoluble organic matter (S4) is weakly represented in these samples, accounting only for 0.60 mg.g^−1^ sample.

These results show that, after 6 months of mineralization at 4 °C, fresh materials (free hydrocarbons, S1) are under-represented compared to more altered compounds. These “old” compounds could be the result of cell lysis and/or extracellular material export over time and may have self-organized into insoluble complexes like humic acids, rendering them more refractory^45^. The evidence for increasing maturity is strengthened by the S3 value, which indicates degradation of oxydized and/or oxygenated hydrocarbons. However this maturity is all relative, as demonstrated by the low level of S4 refractory fraction. This underlies the important effects of diagenesis and metamorphism on the fate of organic molecules preserved in mineral cement: relatively clement and moderate diagenetic conditions in the sedimentary environment will ensure better chemical preservation of *Yersinia* fossils on longer geological timescales. Conversely, more pronounced environmental diagenetic/metamorphic conditions will enhance the degree of degradation and, hence, maturity of the organic molecules.

The potential for molecular preservation within sediments during diagenesis notably depends on the chemical compositions of lipids^45^, especially in terms of unsaturated and aromatic groups. Moreover, it is well known that microorganisms respond to environmental changes by adapting the composition of their cell-wall and membrane lipids^46^. To determine whether the physiological status of *Yersinia* changes the global lipid composition, thus influencing the potentials for molecular preservation on geological timescales, we used GC-MS to characterize and quantify free lipids (S1 fraction) within cells exposed or not exposed to desiccation and radiation stresses (Table 3).

**Table 3:** Relative abundance of *Yersinia* fatty acids detected by GCMS analysis, after 1 month and 6 months of mineralization in silica (Si) and gypsum (Gy), either for cells exposed to dessication +X-rays, or to non-stressed *Yersinia*.

Quantity in µg	T_0_		1 month				6 months			
	Non-stressed	Stressed	Non stressed		Stressed		Non stressed		Stressed	
			Si	Gy	Si	Gy	Si	Gy	Si	Gy
	0.7	1.1	0.8	2	0.8	1.1	1.3	1.4	1.1	0.9
C_12_H_24_O_2_	6.1	4.8	6.8	6.7	4.2	5.2	7.1	6.2	6.4	7.8
C_13_H_26_O_2_	5.4	5.4	5.6	6.5	4.1	4.1	6.5	6.8	5.13	4.9
C_14_H_28_O_2_	7.1	4.6	6.9	7.2	5.1	5.6	5.4	6.5	6.2	7.4
C_15_H_30_O_2_	6.6	4.5	6.5	7.1	4.8	4.8	4.9	6.2	5.1	5.9
C_15_H_30_O_2_ iso	0.5	0.6	0.7	0.9	0.5	1.3	1.0	1.0	0.8	0.8
C_15_H_30_O_2_ antéiso	1	1.4	1.1	1.2	1.2	1.0	0.5	0.6	1.5	1.9
C_16_H_32_O_2_	30.1	26.6	28.2	25.5	28.1	22.0	25.5	28.1	28.9	31.2
C_17_H_34_O_2_	7.2	4.3	6	5.4	7.6	7.7	6.6	3.5	4.5	3.6
C_17_H_34_O_2_ iso	0.8	0.9	1	1.9	1.2	0.8	1.3	0.9	0.855	0.9
C_17_H_34_O_2_ antéiso	0.8	0.7	0.8	1.2	1.9	0.8	0.5	0.5	0.76	1
C_18_H_36_O_2_	31.2	28.5	26.1	24.9	28.1	29.1	26.9	26.9	26.1	21.4
Acide octanedioique	0.2	1	1	0.9	1.3	1.2	1.1	1.4	0.8	0.4
Acide nonadioique	1.8	2.1	1.2	1.7	4.6	5.8	3.2	4.1	2.1	2.5
Octadecenoic acid	1.2	6	8.1	8.9	5.8	8.0	9.4	7.2	7.5	6.7
Traumatic acid (C12 -en-dioic)	ND	8.6	ND	ND	1.5	2.5	ND	ND	3.3	3.5

ND = Not Detected.

Overall, the nature and quantity of free lipids we could quantify were very similar in all experimental conditions (variable time, stress and type of mineral encrustation) and there was no overall change in their biochemistry which could indicate better potential for biomolecule preservation. Between 0.7 and 1.4 µg of lipids were quantified, mostly represented by membrane fatty acids from dodecanoic (C12) to octodecanoic acid (C18). Unsurprisingly, hexadecanoic (C16) and octodecanoic (C18) acids were the two major membrane fatty acids, common in bacteria. These fatty acids could result from the breakdown of membrane phospholipids during acidic hydrolysis (see Materials and Methods), releasing fatty acids as well as glycerol and phosphates that were also detected by GCMS.

Two minor molecular changes could nevertheless be observed. Traumatic acid (dodec-2-enedioic acid) was only detected in the desiccated and irradiated cells, suggesting that this molecule may be produced in response to a single or combined physiological stress. Since traumatic acid was also detected in stressed cells before mineralization (stressed and T_0_ in the Table 3), this molecule could be rapidly produced by stressed cells and remained detectable with time, without being continuously produced.

The relative abundance of octadecenoic acid increased with the duration of mineralization independently of the presence or absence of stress, thus demonstrating that desiccation and radiation cannot explain this increase. Interestingly, the concentration of this lipid has previously been shown to increase after bacterial exposure to various stresses^47^. Since vials for mineralization were kept 6 months at 4 °C without carbon sources, this increase could be due to cold conditions and metabolic constraints, which also represent a stress from a microbial point of view.

However, due to the low relative abundance of these lipids (8.6% for traumatic acid and 8.9% for octadecenoic acid, Table 3), which all together constitute only a part of the S1 fraction, it is unlikely that these minor changes would affect the potential of biogenic lipid preservation over billions years.

The absence of a clear tendency related to the various experimental conditions was confirmed by statistical analysis of lipid distributions using principal component analysis (PCA). Indeed, only low values of correlation matrix were obtained with lipids (highest correlation matrix 0.61 with C12 and C14) and the distribution of samples within the two first components of PCA (explaining only 52% of variability) did not enable to distinction between sample populations, showing that no “stress”, “mineral” or “time” effects could explain the distributions of fatty acids (Supplementary Figure 3 and Supplementary Table 1).

## Discussion

### Biosignature detection during early mineralization

The early entombment of microorganisms within minerals has been shown to strongly increase the preservation of cells during ageing^21, 22, 48^. Since not all microorganisms are susceptible to early mineralization^11, 14^, we first determined whether our *Yersinia* model can be embedded in a mineral matrix or not.

### Stressed Vs non-stressed cell mineralization

One of the goals of this study was to assess potential changes in the nature of biosignatures and their detection when stressful environmental conditions are encountered by cells. No difference in the morphological aspects (embedding of cells in minerals) nor in the spectroscopic/biochemical aspects (RAMAN/FTIR spectroscopy) of mineralization were observed. The only difference concerns minor changes in the relative abundance of octadecenoic acid and traumatic acid, probably not significant enough to impact the preservation of biosignatures on geological timescales. On Mars, although changes in planetary conditions occurred very progressively on the long term, these results do not suggest any improvement in life detection, for microbial physiologies similar to that one of *Yersinia*.

### Morphology and viability of cells

In this study, the precipitation of minerals around *Yersinia* cells increased progressively during 6 months and the morphology of *Yersinia* was preserved in both minerals. This time-dependence of mineralization associated with the preservation of cell morphology has also been demonstrated in previous experimental microbial silicification experiments^10, 12, 14, 21, 49^. However, whereas silica precipitation was rapid and entirely embedded cells, it was slow in the case of gypsum with very local cell/mineral interactions. Moreover, in the case of *Yersinia*, silicification was restricted to the outside of cells while with gypsum we could observe evidence of mineralization within the cell. These differences suggest specificities in *Yersinia*-mineral interactions. We cannot exclude possible replacement of cytoplasm content by silica, *i*.*e* permineralization of *Yersinia*, on longer times, since previous studies reported the influence of the duration of silicification on cultures^49^ and in natural environments^50^.

Three different types of mineralization have been described: Biologically Controlled Mineralization (BCM, for example magnetotactic bacteria), Biologically Induced Mineralization (BIM) and Biologically Influenced Mineralization (BIFM)^19^. It is likely that silicification of *Yersinia* occurs passively through BIFM with the chemical affinity of the mineral to functional groups in its cell-wall occurring through electrostatic interactions and hydrogen bonding, as described previously^7, 13, 14, 50^. Microbial silicification was shown to increase in the presence of biofilms, EPS or sheaths^11, 13, 17, 51^. In this study, none of these components was observed, suggesting that the physiological properties of *Yersinia* do not favor its mineralization.

The preservation of microorganisms in gypsum has been described in natural environments^52^, either in recent evaporites reporting “fresh” eukaryotic and bacterial cells or on older deposits (5 to 6 My)^53^. It showed that communities embedded in evaporites can impact their environment by imprinting their past activity in minerals^54, 55^. On the contrary, the mineralization of *Yersinia* in our liquid gypsum-saturated solution was very slow. This not only suggests that the chemical affinity of sulfate salts to the cell wall was low but also that evaporation of water, as occurs in salty and arid environments promoting precipitation of gypsum and leading to evaporites, is important to efficiently embed cells in gypsum.

Previous studies have described microbial viability and, even, activity^13^, during silicification, notably physiological changes like EPS production, adhesion to substrates or antibiotic resistance. This shows that at the cellular level mineralization can be considered as a chemical stress^11, 13, 34^. The induction of ABC transporter genes during silicification also revealed that cells respond to mineralization by changing the expression of their genome and adapting membrane exchanges^34^. Our MPN results also showed that *Yersinia* populations remained viable during mineralization, both in silica and gypsum, and with or without initial exposure to desiccation and radiation. Although the response of *Yersinia* to these stresses had no visible effect on its morphology, it can be assumed that cells responded to these conditions at the gene expression level, even though we do not have evidence for this. In an environmental context, 0.01% viability corresponds to 10^2^ viable cells.ml^−1^ (cell density in vials is 10^6^ cells.ml^−1^), which could thus recolonize the environment when more favorable conditions are encountered. Thus, although stressful for cells, the progressive mineralization of *Yersinia* does not lead to the mortality of its population after 6 months. This important observation has physiological (response of cells to mineralization) as well as ecological implications (possible initiation of growth in more favorable conditions).

Silica and gypsum are widely present at the surface of Mars^56^. The mineralization of potential Martian microbial populations in silica or in gypsum could have occurred rapidly, without preventing the growth of the remaining viable cells in more favorable conditions, as occurs on Earth today in terrestrial siliceous hot springs^51^. Although we observed higher efficiency of mineralization in silica than in gypsum, these initial observations are mitigated by the fact that (i) the fate of cell remains does not only depend on early embedding in minerals and (ii) microbial cells preserved in gypsum have been often documented on Earth^53–55^. The latter point highlights the importance of evaporation and rapid dehydration events within environments to preserve cells in evaporites and fluid inclusion within the evaporites. Such evaporation events occurred on Mars during the late Noachian period, illustrated for instance with evaporites of the *Meridiani Planum* region^57, 58^.

### Spectroscopic detection of biological molecules

The morphological preservation of cells and their viability after 6 months suggest that biomolecules were preserved during mineralization. However, when compared to the positive controls which showed typical biological signals (Figs 2a and 3a), FTIR and RAMAN spectra of mineralized *Yersinia* lost most of these biosignatures (Figs 2b and 3b and c). Although the embedding of cells within minerals could explain the low detection of the biomolecules, it is likely that the vibration emission of minerals largely overprints the emission of the biomolecules, preventing their detection since vibrational spectroscopy is a non-quantitative method and is very sensitive to resonance phenomena.

Other studies previously reported the difficulty to detect fossilized biomolecules with RAMAN, like the restricted detection of minerals in samples containing microorganisms^22, 23, 54^ or fluorescence emission inhibiting the detection of biomolecules^21^, highlighting thus the importance of resonance pigments^59^.

FTIR results showed that three regions associated the presence of biomolecules could nevertheless still be detected, in agreement with the positive detection of organics in microfossils using FTIR^22^.

These results point out (i) that FTIR and RAMAN spectroscopy are better adapted to the characterization of minerals but much less to the detection of organics and (ii) the importance of using alternative methods to detect organochemical biosignatures in the geological record, such as scanning transmission X-ray microscopy (STXM) and near edge X-ray absorption fine structure (NEXAFS) spectroscopy, two methods that successfully characterized organics in natural and artificial fossils^5, 19, 22, 23, 46, 60^.

The limitations in using RAMAN spectroscopy to detect organics in minerals and rocks, and more especially on Mars, have been previously discussed^61^ and is strengthened by our results. Moreover, considering the difficulty to detect any organics in samples with cell densities as high as ~10^6^ cells.ml^−1^, detecting past traces of life using RAMAN within very low biomass samples, as may have occurred in the Noachian, may be very challenging.

### Antigen detection with SOLID

Proteome modifications due to the regulation of genome expression during the silicification of cells^34^ show the need to use universal antibodies that enable detection of constantly-expressed membrane proteins. Using such antibodies, we observed a strong decrease of fluorescence and the difficulty to detect biomarkers of mineralized cells (Fig. 4).

The lysis of sensitive microorganisms during silicification was not observed in our study and thus cannot explain these results. Moreover, Picard *et al*.^23^ also reported the preservation of proteins during mineralization of cells.

Since not all cells were mineralized after 6 months (Supplementary Figure 1), the embedding of cells with minerals is not sufficient to explain the decrease in fluorescence. We might explain that by (i) the three-dimensional modifications of protein structures, their unfolding due to cold, oligotrophic and mineral-rich conditions during storage, (ii) the high concentration of minerals leading to interactions between minerals and chips and a decrease of sensitivity of the Chip.

The successful detection of mineralized cell biomarkers with SOLID-Chips varies between studies and seems to depend on overall experimental conditions. On the one hand, some studies described the positive detection of biomarkers with SOLID-Chip in mineral-rich environments^43, 44^. On the other hand, other studies highlighted the influence of mineralogical composition on SOLID-Chip sensitivity, with inhibition by clay but not by iron-rich minerals^42^.

Since the embedding of *Yersinia* in a mineral matrix prevents biomarker detection with the SOLID-Chip long before its preservation in the fossil record, the degradation of the functional groups of sugars and proteins that occur on geological timescales, experimentally confirmed during artificial ageing of cells^23^, makes the detection of fossil biomarkers using SOLID-Chips very challenging. From a Martian point of view, although aminoacids are rapidly degraded on the surface of Mars by radiations^62^, the protection of aminoacids in rocks at the Martian subsurface^63^ and the recent description of amide groups preserved in 1.88 Ga old microfossils^60^ suggest that the detection of proteins in a subsurface fossil record on Mars may still be possible.

### Preservation and detection of *Yersinia* microfossils over geological timescales

#### Chemical preservation and Rock-Eval analysis

In combination with other methods, such as GCMS, Rock-Eval analysis is very helpful in detecting past biological activities within sediments. It enabled for example to detect global events on the Earth, such as mass extinctions^64^ and to relate sediment maturity with species-specific biomarkers, thus aiding to reconstruct paleoenvironments millions of years old^64^.

Our results showed that the TOC of mineralized *Yersinia* was low and that fresh and free molecules (S1 fraction) dominated insoluble materials (Table 2). Although more refractory compounds were present (S3 fraction), they were lower in abundance and the most refractory fraction (S4) was extremely under represented. The low level of TOC also makes detecting organic compounds difficult. The fate of these insoluble molecules thus strongly depends on environmental conditions encountered during diagenesis. Indeed, clement and gentle temperatures, pressures and non-oxidizing conditions are needed to prevent the total degradation of the dominant but sensitive S1 fraction. However, in the case of more drastic diagenetic conditions, S1 and S2 fractions will be totally degraded and the small S4 fraction would be detectable after billions years.

Although globally rare, previous descriptions of microbial traces in Archean rocks^3, 5^ demonstrate the possibility to meet favorable conditions for chemical fossil preservation over billions of years in natural environments. For instance, Westall *et al*.^5^ used XANES (X-ray Absorption Near Edge Structure) at the sulfur K-edge to document the presence of thiophene, a signature of organic matter degradation by sulfur reducing bacteria, in rocks more than 3 billion years old, as well as *in situ* ToFSIMS to determine the range of degraded organic molecules (mostly aromatic) whose restricted composition (as opposed to the broad compositions found in meteorites, for example) indicates a biological origin.

In all scenarios, we can expect that the degradation of molecules presenting functional groups will occur first, especially the amide/amine and hydroxyl groups of proteins and sugars that we detected by RAMAN and FTIR spectroscopy.

#### Artificial ageing of samples

The artificial mineralization of microorganisms, as reported in this study, does not provide information about the degradation of biosignatures during long-term diagenesis and metamorphism. Minor changes in amino and fatty acid compositions during one year of silicification of *Archeae* ^15^ illustrates that mineralized cells cannot be considered as true microfossils, here from a biochemical point of view. To overcome this, mimicking geological time by artificially ageing samples after exposure to high temperature and pressure conditions to better understand the morphological, chemical and physical modifications of biosignatures with ageing began as early as 1976^65^, and similar studies were published more recently^19, 21–23, 48, 60^.

An important conclusion emerging from these studies is that preservation of cells is enhanced by their embedding in mineralogical matrices, reported either in silica^22, 65^ or in calcium carbonate^21^. Picard *et al*.^22^ documented that silica had limited influence to preserve cells since, in this study, iron oxides produced by iron-oxidizing bacteria attached to cell-walls and protected cells, thus substituting the preserving effect of silica. The role of Fe-phases and cation bridges in mineralization, elsewhere reported^12^, highlights the critical influence of mineral composition to preserve cells in their rocky environment.

In our case, the efficient mineralization in silica of *Yersinia* suggests that early silicification may strongly favor its preservation in the rock record, as reported in the previous ageing studies mentioned above. Crystallization of Opal-A to quartz improved chemical preservation of photosynthetic microorganisms^48^, suggesting that similar effects could be expected with the Opal-A silica associated to *Yersinia*.

However, the potential for long-term preservation may depend on the type of mineral since the low rate of *Yersinia* mineralization in gypsum (Fig. 1) indicates that such protective effects might be limited, at least with this model. The absence of metabolism based on iron-oxidation with *Yersinia* means that this step of early mineralization is even more important, in contrast to organisms like *Acidovorax sp*. BoFeN1, which could benefit of iron-phase protection without silica^22, 23^. In addition, the importance of extracellular structures for early mineralization suggests that *Yersinia* physiology do not favor its preservation.

Concerning chemical preservation, sugars or lipids of *Yersinia* may be detected with diagenesis since our FTIR results showed these signals using FTIR (C-O, C-C, C-OH, C-O-CCH, CH_2_/CH_3_ signals, Fig. 2b). The D and G bands of disordered carbon may also be detected with Raman, as already reported in Alleon^48^ and in ancient carbonaceous microfossils^6^.

**Figure 5:**
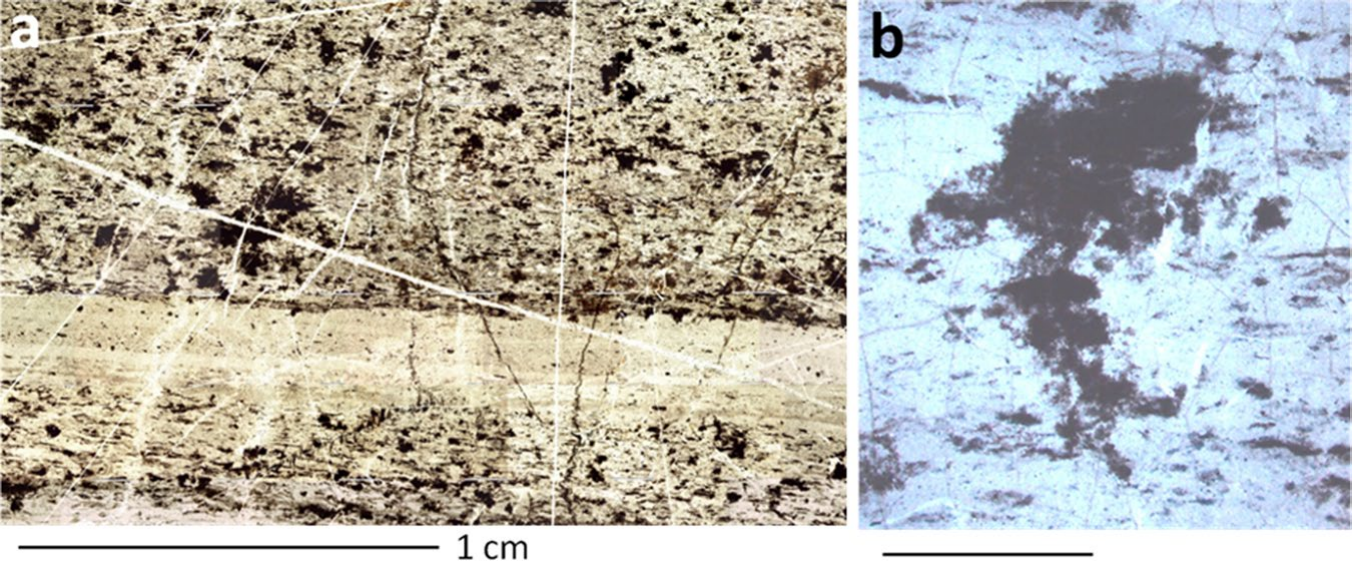
Hydrothermal chert facies from the 3.33 Ga-old Josefsdal Chert, Barberton Greenstone Belt, South Africa^6^; (**a**) large-scale photomicrograph showing layered concentrations of disseminated carbonaceous clots interpreted as possible heterotrophic colonies; (**b**) details showing one of the clots with a “spiky” three dimensional morphology, having no relationship to the bedding plane layers, indicating *in situ* growth.

In the absence of extracellular sheaths and iron-based metabolism, lyses of *Yersinia* cells will lead to the release of bacterial remains and materials and thus on the widespread dissemination of organic matter in rocks. Individual cells may have lysed, but the colony morphology could remain intact, as documented by fossilized carbonaceous remains in natural samples, such as low grade metamorphosed hydrothermal cherts of the Archean^6^ (Fig. 5).

All artificial ageing studies mentioned above were made in a terrestrial context, whereas better understanding of potential traces of life on Mars requires reproducing metamorphic conditions closer to a Martian context. On Mars, the absence of plate tectonics, the lower mantle internal activity and lower subsurface pressures compared to Earth, all lead to a lower degree of metamorphism and of diagenesis (although important regional variations should be envisaged due to local volcanic and/or hydrothermal activity and/or impact shock). This implies that temperature and pressure values applied to artificially age these samples may be lower, and reduced degradation of organics could be expected. However, to age fossilized samples in relevant Martian conditions, modeling the evolution of temperature and pressure in the Martian subsurface is required.

### Concluding remarks

In this investigation, we experimentally mineralized a bacterial model, isolated from an Early Mars analogue environment and studied its potential for preservation on diagenetic conditions. While no difference was observed between cells exposed or not exposed to typical Martian stresses, differences in potential for fossilisation were observed depending on the nature of encasing minerals. Although rich in organics, some methods (FTIR) are more efficient than other (RAMAN and SOLID-Chips) in detecting biomarkers after mineralization in our samples. The biogeochemistry of our mineralized *Yersinia*, as revealed by Rock-Eval analyzes, implies that preservation of such microfossils in the geological record can be expected only if a low degree of metamorphism is encountered by the mineralized cells^38, 41^.

## Electronic supplementary material

Supplementary information
