# Supplementary material for: Mineralization and Preservation of an extremotolerant Bacterium Isolated from an Early Mars Analog Environment
Source: Sci Rep. 2017 Aug 18;7:8775. doi: 10.1038/s41598-017-08929-4 (PMC5562696; doi:10.1038/s41598-017-08929-4)
Supplement: Supplementary file 1 — Supplementary information [file 41598_2017_8929_MOESM1_ESM.pdf]

MINERALIZATION AND PRESERVATION OF AN EXTREMOTOLERANT BACTERIUM ISOLATED FROM AN EARLY MARS ANALOG ENVIRONMENT

Gaboyer F*[1], Le Milbeau C[2,], Bohmeier M[3], Schwendner P[4], Vannier P[5], Beblo-Vranesevic K[3], Rabbow E[3], Foucher F[1], Gautret P[2], Guégan R[2], Richard A[6], Sauldubois A[6], Richmann R[2], Perras AK[12,13], Moissl-Eichinger C[7], Cockell CS[4], Rettberg P[3], Marteinsson V[5], Monaghan E[8], Ehrenfreund P[8], Garcia-Descalzo L[9], Gomez F[9], Malki M[10], Amils R[10], Cabezas P[11], Walter N[11] and Westall F[1]

[1] Centre de Biophysique Moléculaire, CNRS, Orléans, France. frederic.gaboyer@cnrs-orleans.fr

[2] Institut des Sciences de la Terre d'Orléans, UMR 7327, CNRS-Université d'Orléans, 1A Rue de la Férollerie, 45071 Orléans Cedex 2, France

[3] Institute of Aerospace Medicine, Radiation Biology Department, German Aerospace Center (DLR), Cologne, Germany

[4] UK Center for Astrobiology, School of Physics and Astronomy, University of Edinburgh, Edinburgh, United Kingdom

[5] MATIS - Prokaria, Reykjavík, Iceland

[6] Centre de Microscopie Electronique, Université d'Orléans, Orléans, France.

[7] BioTechMed Graz, Graz, Austria

[8] Leiden Observatory, Universiteit Leiden, Leiden, Netherland

[9] Instituto Nacional de Técnica Aeroespacial – Centro de Astrobiología (INTA-CAB), Madrid, Spain

[10] Universidad Autónoma de Madrid (UAM), Madrid, Spain

[11] European Science Foundation (ESF), Strasbourg, France

[12] University Regensburg, Department of Microbiology, Regensburg, Germany

[13] Medical University of Graz, Department of Internal Medicine, Graz, Austria

Supplementary information

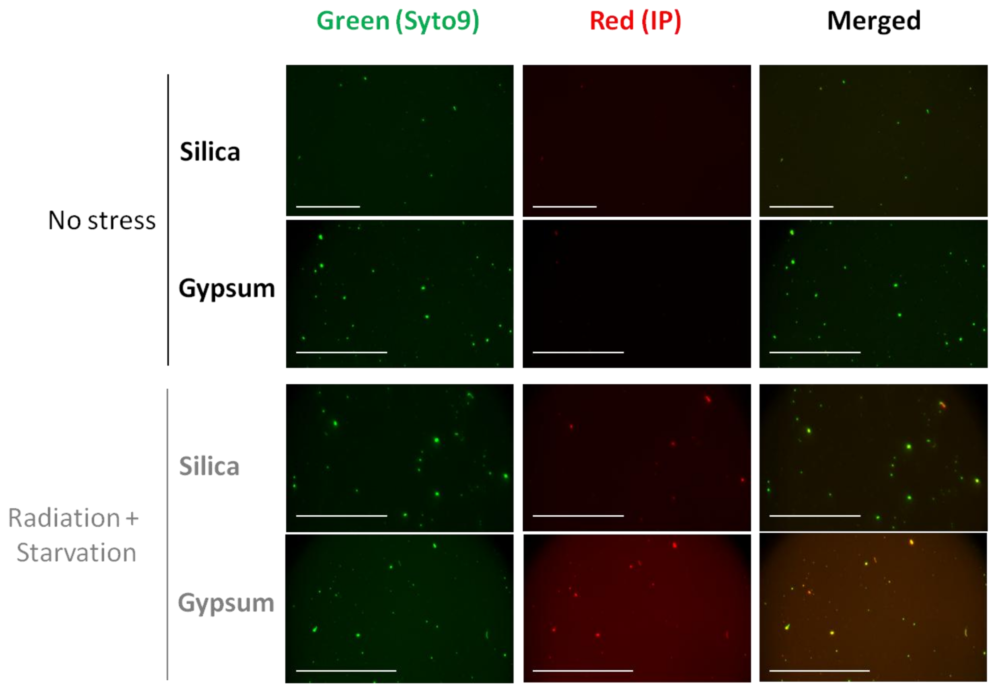

Supplementary Figure 1 : Images of *Yersinia* cells after 6 months of mineralization stained with the Live/Dead© stains. The figure shows the Syto9 fluorescence only of “intact” cells (left part), the IP fluorescence only of “permeabilized” cells (middle part) and the merged images (right part) for both cells non-exposed (upper part) and exposed (lower part) to desiccation and radiation stresses. Scale bars : 50µm.

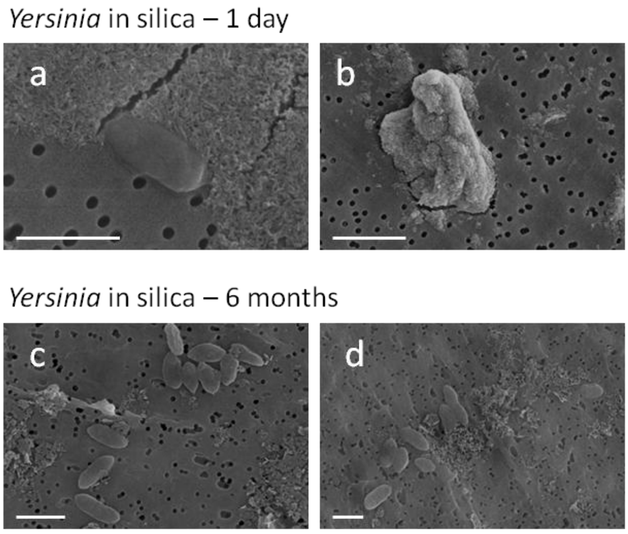

Supplementary Figure 2 : SEM images of *Yersinia* showing that silicification of cells is rapid (a-b) and heterogeneous within the population (c-d). (a-b) *Yersinia* after 1 day of silicification showing that cells are rapidly in close contact to precipitated silica. (c-d) Images of *Yersinia* after 6 months, revealing several cells free of silica and thus that mineralization of cells is heterogeneous and varies within the population. Scale bars : 1µm.

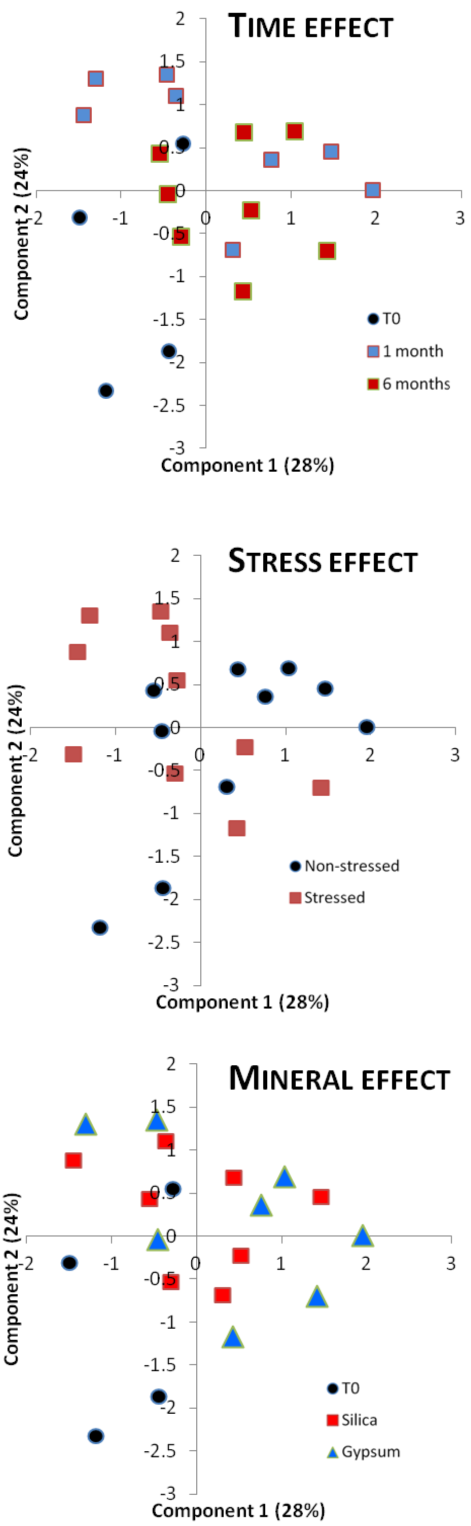

Supplementary Figure 3 : Principal component analysis of samples with the two first components, as a function of time, stress and mineral, as well as the distribution of variables. This shows that no clear "stress', "mineral" or "time" effects enables to distinguish between sample populations. The PCA analysis was done using the KyPlot software for 20 samples and 14 variables (fatty acids concentrations). The first two principal components accounted for 52% of the total variability, with respectively 28% for component 1 and 24% for component 2 (indicated in brackets on axis legends).

Supplementary Table 1: Correlation matrix (KyPlot software) of the relative abundance of fatty acids used as variables in the principal component analysis, showing that no tendency related to the experimental conditions was (highest correlation matrix 0.61 with C12 and C14). Traumatic acid, also detected in samples from our different experimental conditions (stress and no stress, silica or gypsum, T0, 1 month or 6 months) was excluded because of its clearly bimodal distribution (present in stressed samples, absent in non-stressed ones).

	C12H24O2	C13H26O2	C14H28O2	C15H30O2	C15H30O2 iso	C15H30O2 anteiso	C16H32O2	C17H34O2	C17H34O2 iso	C17H34O2 anteiso	C18H36O2	Octanedioic acid	Nonanedioic acid	Octadecenoic acid
**C12H24O2**	1													
**C13H26O2**	0,48	1,00												
**C14H28O2**	0,62	0,22	1,00											
**C15H30O2**	0,56	0,42	0,61	1,00										
**C15H30O2 iso**	0,23	0,31	0,05	0,09	1,00									
**C15H30O2 anteiso**	0,15	0,24	0,39	0,31	0,05	1,00								
**C16H32O2**	0,17	-0,21	0,16	0,06	-0,48	-0,37	1,00							
**C17H34O2**	-0,23	-0,31	-0,10	-0,21	-0,15	0,06	-0,43	1,00						
**C17H34O2 iso**	0,14	0,38	-0,16	0,35	0,38	0,16	-0,42	0,07	1,00					
**C17H34O2 anteiso**	-0,10	-0,13	0,02	0,13	0,16	0,49	-0,10	0,23	0,28	1,00				
**C18H36O2**	-0,59	-0,34	-0,45	-0,41	-0,51	-0,61	0,18	0,30	-0,22	-0,43	1,00			
**Octanedioic acid**	-0,36	0,16	-0,41	-0,38	0,34	0,22	-0,55	0,06	0,22	0,16	-0,20	1,00		
**Nonanedioic acid**	-0,52	-0,35	-0,39	-0,53	0,28	-0,29	-0,33	0,39	-0,10	0,07	0,22	0,49	1,00	
**Octadecenoic acid**	0,24	0,36	-0,13	-0,09	0,39	0,14	-0,52	-0,05	0,38	0,05	-0,50	0,57	0,04	1,00
